# Arhovin Internally in Acute and Chronic Gonorrhea

**Published:** 1909-12

**Authors:** 


### ABSTRACTS.

---

### Arhovin Internally in Acute and Chronic Gonorrhea.

STAFF Physician Knauth, Surgeon of the Second Train Batallion (*Muenchen. med. Wochenschrift,* April 21, 1908), published from the Army Post Hospital at Wuerzburg, Germany, "A Contribution to the Internal Use of Arhovin in Acute and Chronic Male Gonorrhea." The many favorable reports regarding arhovin incited him to request permission from the authorities to experiment with the drug. He has in the past year treated twenty-nine cases of gonorrheal disease with arhovin—namely, eleven acute gonorrheas, eleven subacute or chronic gonorrheas, and seven gonorrheal epididymites. The patients received daily four to six arhovin capsules per os, and during the first acute inflammatory stage were kept in bed, on a bland diet—chiefly milk—and with topical ice applications. Only when the discharge had become somewhat more sero-mucus did the patients become ambulant, resuming ordinary diet but without alcohol.

In the chronic forms the patients received the ordinary hospital diet, arhovin being administered in the same dose till the discharge ceased, the urine was clear and free from gonococci, and the inflammatory manifestations from the epididymis had retrogressed. In all cases, even those with sensitive stomachs, arhovin was well tolerated; disturbances of the digestive organs, which might have been expected from the oily consistency of the remedy, were never complained of. Nor were exanthemas or renal irritations ever observed. Arhovin hence has proved to be wholly harmless.

In the most acute stage the disagreeable and at times distressing symptoms, as urinary tenesmus, burning urination, painful erections, were influenced extraordinarily favorably. They usually become milder in the first night and, at the latest, disappeared definitively after the third day in the hospital. Not only did the first stormy symptoms pass rapidly, but the further course of the process was also almost always visibly shortened. The average length of treatment in the acute cases was thirty days, and in five cases cure ensued within two to three weeks.

The internal arhovin treatment was also successful in chronic forms of gonorrhea; average duration of the disease was forty days, and of the eighteen treated only one suffered a relapse. The others must be considered cured, since they have passed the monthly medical inspections. Arhovin acts most favorably on the inflammation of the urethral mucosa in the most acute stage, allaying the pain. It shortens the course of the disease and lessens or prevents the dangerous complications.

---

